# Potential of immunomodulatory agents as adjunct host-directed therapies for multidrug-resistant tuberculosis

**DOI:** 10.1186/s12916-016-0635-1

**Published:** 2016-06-15

**Authors:** Alimuddin Zumla, Martin Rao, Ernest Dodoo, Markus Maeurer

**Affiliations:** Division of Infection and Immunity, University College London, and NIHR Biomedical Research Centre, UCL Hospitals NHS Foundation Trust, London, UK; F79, Therapeutic Immunology (TIM) division, Department of Laboratory Medicine (LABMED), Karolinska University Hospital Huddinge, 14186 Stockholm, Sweden; Centre for Allogeneic Stem Cell Transplantation (CAST), Karolinska University Hospital Huddinge, Stockholm, Sweden

**Keywords:** Tuberculosis, Multidrug resistance, Host-directed therapies, Immunomodulatory agents, Immune response, Inflammation

## Abstract

Treatment of multidrug-resistant tuberculosis (MDR-TB) is extremely challenging due to the virulence of the etiologic strains of *Mycobacterium tuberculosis* (*M. tb*), the aberrant host immune responses and the diminishing treatment options with TB drugs. New treatment regimens incorporating therapeutics targeting both *M. tb* and host factors are urgently needed to improve the clinical management outcomes of MDR-TB. Host-directed therapies (HDT) could avert destructive tuberculous lung pathology, facilitate eradication of *M. tb*, improve survival and prevent long-term functional disability. In this review we (1) discuss the use of HDT for cancer and other infections, drawing parallels and the precedent they set for MDR-TB treatment, (2) highlight preclinical studies of pharmacological agents commonly used in clinical practice which have HDT potential, and (3) outline developments in cellular therapy to promote clinically beneficial immunomodulation to improve treatment outcomes in patients with pulmonary MDR-TB. The use of HDTs as adjuncts to MDR-TB therapy requires urgent evaluation.

## Background

The World Health Organization (WHO) presently ranks tuberculosis (TB) as the leading cause of death due to infectious disease worldwide, with an estimated 1.5 million deaths from TB in 2014 [1]. Multidrug-resistant TB (MDR-TB) accounted for more than 3 % of newly diagnosed TB cases, and over 10 % (190,000) of all TB-related deaths [1]. Further, 12 % of individuals diagnosed with TB were also co-infected with HIV, comprising more than 20 % (390,000) of deaths due to TB in 2014 [1]. Current WHO guidelines for MDR-TB treatment recommend the use of combination TB drug therapies that target *Mycobacterium tuberculosis* (*M. tb*). These drugs, several of which have serious side effects, have to be taken for a period of 12–18 months, with the long duration resulting in poor patient compliance. Furthermore, current MDR-TB treatment regimens cure only 50 % of MDR-TB cases and impose a huge financial toll on national health services [1, 2]. Other important limitations of current MDR-TB treatment include drug interactions and insufficient drug concentrations in patients’ lungs [3] as well as aberrant or excess host inflammatory responses to *M. tb*, resulting in lung tissue destruction, compromising the body’s ability to eradicate the *M. tb* bacilli, and leading to permanent lung tissue damage [4–6].

Pulmonary pathology observed in patients with all forms of TB can be attributed to intrinsic exaggerated inflammatory responses to *M. tb* [4]. Since HIV co-infection is the major comorbidity associated with poor clinical outcome in patients with active TB, early restoration of immune cells in HIV-TB patients following antiretroviral therapy (ART) can result in the development of immune reconstitution inflammatory syndrome (TB-IRIS) [5]. In TB-IRIS, existing *M. tb*-specific T cells proliferate uncontrollably, consequently perpetrating local and systemic overt immune reactions and potentially leading to death [5]. An exaggerated release of pro-inflammatory cytokines, such as tumour necrosis factor alpha (TNF-α), interleukin 6 (IL-6), IL-8, IL-18, and interferons alpha and beta (IFN-α/β), invariably perpetrate tissue damage in active TB as well as TB-IRIS [6, 7]. Although TB-IRIS is more closely associated with drug-susceptible TB patients starting on ART, it has been reported in South African patients with MDR-TB and HIV co-infection [8]. An exigent need therefore exists to develop new strategies to treat and prevent harmful inflammatory responses in MDR/extensively drug resistant-TB patients to avoid long-term, if not permanent, lung damage [9–12].

Considering these limitations and intricate clinical scenarios in TB, host-directed therapies (HDTs) have emerged as a new and promising intervention avenue against all forms (drug-sensitive and drug-resistant) of the disease [13]. HDTs generally target clinically relevant biological pathways in the host to modulate and rectify pathological immune responses. In TB, HDTs may neutralise excessive inflammation in organs and decrease *M. tb* proliferation while facilitating tissue repair [7, 10].

## Therapeutic targeting of immunological and cellular processes in MDR-TB

**Fig. 1:**
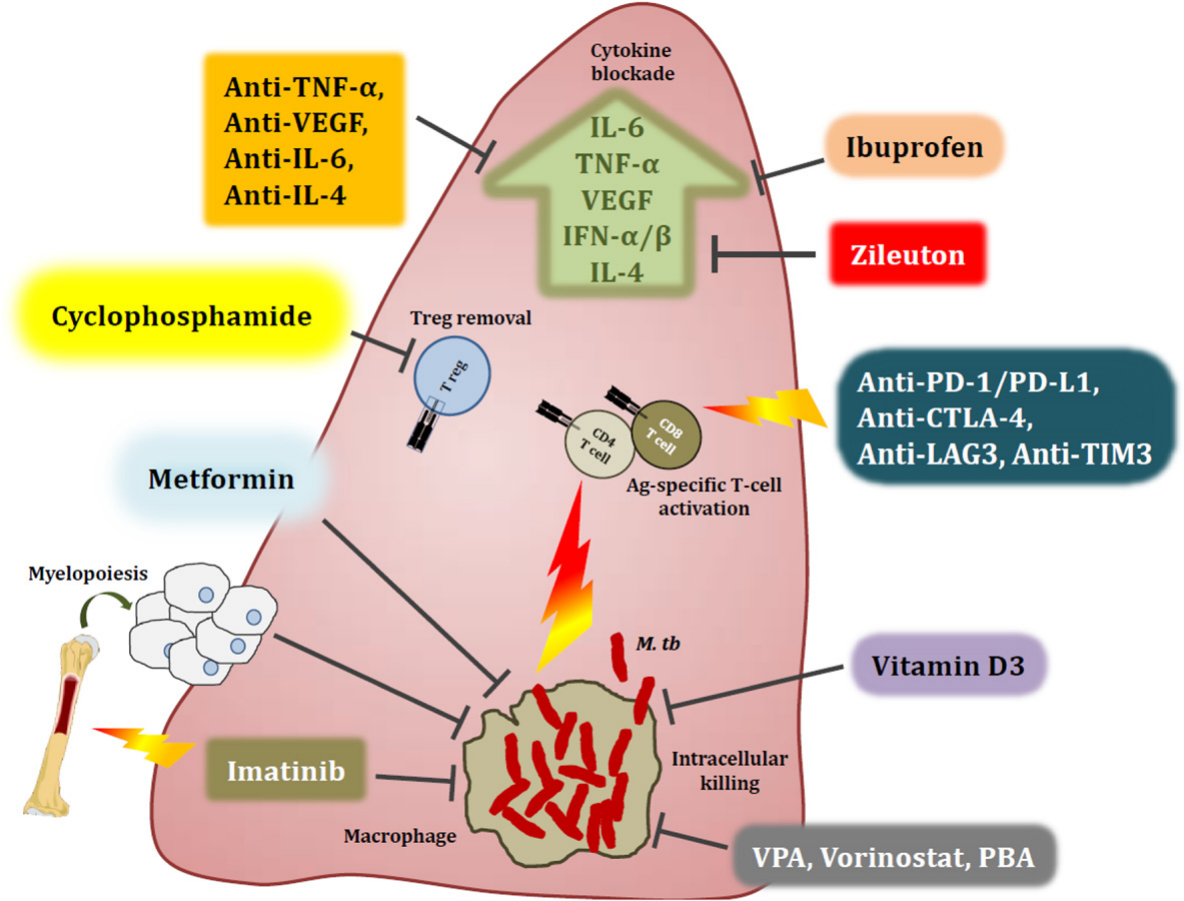
Host-directed therapies aimed at modulating immune responses in the tuberculous lung. Overt immune responses characterise the pathological outcome in tuberculosis (TB). Neutralisation of pro-inflammatory cytokines such as IL-6, TNF-α, VEGF and IFN-αβ, as well as anti-inflammatory IL-4 during severe pulmonary disease may help reduce ongoing parenchymal damage in the lung. Alternatively, suboptimal activation of anti-TB immune responses due to regulatory T cell activity can be reversed by the use of the anti-cancer drug cyclophosphamide. Drugs with anti-TB potential, such as metformin, imatinib, ibuprofen, zileuton, valproic acid, and vorinostat as well as nutraceuticals such as vitamin D3 not only abate bacterial burden via host-dependent mechanisms, but may also fine-tune the immune response to *Mycobacterium tuberculosis* (*M. tb*). These drugs increase phagocytosis of extracellular bacteria, improved emergency myeloid response and increased autophagic and apoptotic killing of bacteria, subsequently editing the T cell response in favour of the host. Immune checkpoint inhibition with blockade of the PD-1/PD-L1, CTLA-4, LAG3 and TIM3 pathways may improve the quality of the cellular immune response to *M. tb* epitopes, as seen in cancer. A more complete list of currently pursued host-directed therapies for TB can be found in Table 1. Abbreviations: VPA, valproic acid; PBA, phenylbutyrate; PD-1, programmed cell death 1; PD-L1, PD-1 ligand 1; CTLA-4, cytotoxic T lymphocyte-associated antigen 4; LAG3, lymphocyte-activation gene 3; TIM3, T cell immunoglobulin and mucin domain 3

Over the past several years, extensive preclinical and clinical studies have revealed a myriad of immunological pathways and biomarkers that either influence the outcome of *M. tb* infection or indicate the state of disease, respectively [6, 14–18]. These pathways include cytokine-mediated signalling, intracellular antimicrobial processes, and establishment of long-term immunological memory as well as physiological homeostasis [13]. The use of immunomodulatory agents as HDTs for TB treatment has therefore garnered great interest as a means of improving clinical outcome of therapy, based on previous experience with other infectious diseases and cancer. Many of these agents are already licensed for non-TB indications, thus bearing US Food and Drug Administration approval, while others are in advanced clinical trials [18, 19]. A summary of the various HDT agents with therapeutic potential in pulmonary MDR-TB is presented in Table 1 and Fig. 1.

**Table 1:** List of immunomodulatory agents for the treatment of multidrug-resistant tuberculosis

Immunomodulatory agent	Host target	Currently licensed indication(s)	Biological activity	Ref.
Small molecules				
Metformin	AMPK activator	Diabetes	Augments mitochondrial reactive oxygen species-mediated intracellular MDR *M. tb* killing and reduction of lung bacterial burden and pathology in mice likely via increased mitochondrial turnover; enhances CD8 T cell responses, possibly by increasing FAO with AMPK involvement in memory cells. Shown to promote anti-tumour CD8 T cell memory generation in engrafted TNF receptor associated factors knockout mice via FAO restoration	[41, 43, 120]
Zileuton	5-lipoxygenase inhibitor	Asthma	Inhibits 5-lipoxygenase and subsequent formation of leukotrienes; promotes reduced lung *M. tb* burden and pathology in mice by increasing PGE2 levels and augmenting IL-1β-mediated anti-TB immune control	[29, 30]
Ibuprofen	COX inhibitor	Pain and fever relief	Inhibits COX2 and suppresses prostaglandin H2 and thromboxane production; inhibits COX1; reduces lung pathology and *M. tb* load in a highly-susceptible TB mouse model	[27]
Aspirin (acetylsalicylic acid)	COX inhibitor	Pain and fever relief	Inhibits COX1 to suppress prostaglandin and thromboxane production to dampen TNF-α-induced overt inflammation; aids tissue repair and control of *M. tb* burden	[121]
Valproic acid	Histone deacetylase inhibitor	Epilepsy and bipolar disorder	Inhibits HDAC I, II and IV to block histone deacetylation and enhance gene transcription; activates latent HIV reservoirs and increases ART efficacy as well as increased CD8 T cell activity; can induce autophagy and apoptosis	[61, 122, 123]
Carbamazepine	GABA receptor agonist and sodium channel stabiliser	Epilepsy and neuropathic pain	Induces autophagy via inositol depletion in macrophages, potentiating killing of intracellular *M. tb*; reduces lung pathology and improves overall immune responses in a mouse model of TB	[124]
Vorinostat	Histone deacetylase inhibitor	Cutaneous T cell lymphoma	Inhibits HDAC I, II and IV to block histone deacetylation and enhance gene transcription; induces reactivation of latent HIV in CD4 T cells and improves CD8 T cell responses as well as ART efficacy – presently in clinical trials in HIV-infected individuals; can induce autophagy and apoptosis; shown to dampen neuroinflammation in a mouse model of West Nile virus infection, adjunctively to an experimental antiviral drug candidate	[62, 69, 125–127]
Phenylbutyrate	Histone deacetylase inhibitor, chemical chaperone	Urea cycle disorders	Inhibits HDAC I to block histone deacetylation and enhance gene transcription; induces autophagy by activating expression of antimicrobial peptides by macrophages to kill intracellular *M. tb* in combination with vitamin D3; shown to be very beneficial as short-course therapy (with vitamin D3) in a clinical study involving patients with pulmonary TB	[70, 71, 127, 128]
Cyclophosphamide	DNA alkylating agent	Lymphomas and pre-transplant preconditioning	Forms lethal phosphoramide mustard following activation specifically in low producers of aldehyde dehydrogenase (largely Tregs); activity shown to potentiate renal cell carcinoma clinical vaccine candidate; Treg depletion may imply clinically beneficial immune responses in severe pulmonary TB	[49, 51, 82, 85, 129]
Etoposide	Topoisomerase inhibitor	Various cancer types	Inhibits DNA topoisomerase I activity to abrogate cell proliferation; depletion of pathogenic inflammatory T cells in influenza-induced hemophagocytic lymphohistiocytosis shown to be beneficial	[130]
Imatinib mesylate	Tyrosine kinase inhibitor	Leukaemias and gastrointestinal stromal tumours	Inhibits mutant BCR-ABL tyrosine kinases in cells; reduces colony forming unit load and pathology in lungs of *M. tb*-infected mice; induces myelopoiesis	[44, 45]
Niraparib	PARP inhibitor	Ovarian and breast cancers	Inhibits PARP1/2 to cause double strand DNA breaks in cells, abrogating proliferation; niraparib has been shown to restore mitochondrial respiration in human muscle fibres, likely by improving FAO, thus promoting maintenance of anti-TB memory CD8 T cells	[131]
Prednisone	Glucocorticoid receptor agonist	Immunosuppressant used in cancer and inflammatory diseases	Activated downstream signalling of the GC receptor; has pleiotropic outcomes, including anti-inflammatory effects; use in community-acquired pneumonia showed improved survival among patients; results in TB patients inconclusive and requires further validation	[20, 22, 132]
*Nutraceuticals*				
Resveratrol	Sirtuin agonist	Over-the-counter antioxidant	Increases cellular mitochondrial turnover, thus increased respiratory capacity; may promote maintenance of anti-TB memory CD8 T cells via FAO increase; alternatively, may also induce apoptosis of activated T cells during severe inflammation	[120, 133]
Vitamin D3	Innate immune response activator	Dietary supplement	Kills intracellular *M. tb*; activates innate immune responses in macrophages, thus improving ensuing T cell responses in combination with phenylbutyrate; also augments IL-32 and IL-15-mediated immune responses in clinical TB	[70, 71, 85, 86, 88, 128, 134]
Biologicals				
Interleukin 15	Involved in CD8 memory T cells maintenance	In clinical trials for various cancers	Signals via IL-15Rβ and the common chain to activate STAT3 and STAT5; increases mitochondrial mass and fatty acid oxidation in memory CD8 T cells to prolong survival and maintenance; augments IFN-γ and vitamin D3-mediated immune responses in human TB	[86, 135, 136], NCT01727076
Nivolumab/pembrolizumab (anti-PD-1)	Immune checkpoint inhibitor	Melanoma; in clinical trials for various other cancers	Inhibits PD-1 expressed on T cells, and abrogates interaction with PD-L1 on tumour cells and myeloid cells to reverse T cell exhaustion increases tumour-specific CD8 T cell activity and tumour regression in metastatic melanoma patients; highly expressed on Tregs isolated from peripheral blood of MDR-TB patients; in vitro blockade of PD-1 on T cells from TB patients potentiated *M. tb* antigen-dependent IFN-γ secretion; anti-TB treatment success is commensurate with lower PD-1 expression in patients	[137–140]
Ipilimumab (anti-CTLA-4)	Immune checkpoint inhibitor	Melanoma; in clinical trials for various other cancers	Inhibits CTLA-4 expressed on T cells, abrogates interaction with CD80 and/or CD86 on tumour cells and myeloid cells to reverse T cell exhaustion; increases CD8 T cell activity and tumour regression in melanoma patients; highly expressed on Tregs isolated from peripheral blood of MDR-TB patients; may potentiate cellular immune responses in clinical TB	[141]
Anti-LAG3	Immune checkpoint inhibitor	In clinical trials for various cancers	Inhibits LAG3 expression; blockade of LAG3 can prevent T1DM development in mice, potentiate CD8 T cell activity of a prostate cancer vaccine candidate and enhance antimalarial immune responses; in non-human primate models of TB, LAG3 expression on CD4 T cells has been shown to correlate with poor anti-TB immune function; blocking LAG3 may contribute to successful containment of TB infection by T cells	NCT02460224, NCT02061761, [106]
Adalimumab (anti-TNF-α)	Cytokine neutralisation	Rheumatoid arthritis	Removes excess TNF-α from systemic circulation and target organs; successfully used as salvage therapy in a patient with severe pulmonary TB	[72]
Siltuximab (anti-IL-6)	Cytokine neutralisation	Juvenile arthritis, Castleman’s disease	Removes excess IL-6 from systemic circulation and target organs; use in MDR-TB patients co-infected with HIV may aid in management of ART-induced TB-IRIS	[6, 74, 142, 143]
Tocilizumab (anti-IL-6R)	Cytokine signalling blockade	Arthritis, Castleman’s disease	Prevents IL-6 from binding to its receptor on cell surfaces to reduce pro-inflammatory signalling; use in MDR-TB patients infected with HIV may aid in management of ART-induced TB-IRIS	[6, 74, 142, 143]
Bevacizumab (anti-VEGF)	Angiogenesis inhibitor	Various cancer types (mostly solid tumours)	Inhibits binding of VEGF-A to its receptor to block signalling and subsequent formation of new blood vessels; bevacizumab inhibited neovascularisation and improved lung pathology in a rabbit model of TB; may also facilitate drug penetration into granulomas and increased oxygenation, with implications for enhancing anti-TB drug efficacy	[79, 144]
Cellular therapy				
Bone marrow-derived mesenchymal stromal cells	Reduction of inflammation and improved tissue regeneration	In clinical trials for various inflammatory indications	Successful phase 1 safety study of mesenchymal stromal cell reinfusion into patients with MDR/extensively drug resistant-TB in Belarus; showed improved lung radiographic findings, pulmonary function (57 % cure); promoted fine-tuning of T cell responses to specific *M. tb* antigens in recipients; trial is currently being repeated in Durban, South Africa	[112, 114, 145]
Antigen-specific T cells	Targeted killing of *M. tb*-infected host cells	Cancer and viral infections	Currently used in cancer immunotherapy; successfully used in treating post-transplantation opportunistic viral infections, i.e. cytomegalovirus, Epstein–Barr virus	[111–113, 115, 116, 118, 146]

*ART* antiretroviral therapy, *IRIS* immune reconstitution inflammatory syndrome, *FAO* fatty acid oxidation, *HDAC*, Histone deacetylase inhibitors, *MDR* multidrug resistant; *M. tb Mycobacterium tuberculosis, TB* tuberculosis

### Immunomodulatory small-molecule drugs

Several non-antibiotic drugs with immunomodulatory properties have already shown clinical efficacy in ameliorating infection-induced pathology. For example, interventional use of glucocorticoid receptor agonists, i.e., prednisone and dexamethasone, has mortality-reducing effects in community-acquired pneumonia, concomitant with lowering of pro-inflammatory cytokine release [20, 21]. However, the clinical outcome of prednisone therapy in pulmonary TB has been inconclusive; further studies are required to ascertain a clear beneficial effect in patients [22].

Eicosanoid regulation dependent on arachidonic acid (ARA) metabolism has been associated with modulation of clinically relevant immune responses and pathology in patients with active TB [23]. Two major arms of enzymatic reactions carry out ARA catabolism: the first involving cyclooxygenases 1 and 2 (COX1 and 2) and the other, lipoxygenases (LO). Breakdown of ARA by COX enzymes progresses to prostaglandin synthesis, while LO metabolise ARA to produce leukotrienes, lipid mediators responsible for fever induction during active infection [24]. COX2 is particularly associated with regulation of pro-inflammatory cytokine production and inflammation via prostaglandin E2 (PGE2) production [25]. Leukotrienes are also involved in initiation of inflammation, angiogenesis and progression of cancer [25].

It has been shown in TB that balance between concentrations of lipoxin A4 (LXA4) and leukotrienne B4 controls subsequent TNF-α release by activated immune cells in the lung [23]. Overproduction of LXA4 may instead cause immunosuppression, highlighting that maintenance of this axis in TB is pivotal in determining patient survival or death. The use of aspirin (acetylsalicylic acid), a non-steroidal anti-inflammatory drug (NSAID) inhibits COX2 activity, thus enhancing LXA4 synthesis, which in turn would decrease TNF-α levels and subsequently neutralise pulmonary inflammation in TB patients [23, 26]. The same may be achieved with ibuprofen (another NSAID), which has been shown to improve survival of experimental mice with severe lung TB by dampening tissue pathology while reducing *M. tb* load [27]. Ibuprofen also has direct anti-*M. tb* activity [28].

Zileuton is an anti-inflammatory drug currently licensed for the treatment of asthma that exerts its effects by inhibiting 5-LO activity [29]. As opposed to NSAIDs, zileuton blocks LO-mediated leukotriene production, facilitating COX2 activation and PGE2 synthesis. In a mouse model of TB, zileuton was shown to dramatically abate IFN-α/β levels in the lung to improve lung pathology, in addition to reducing *M. tb* load [30]. Combination therapy comprising zileuton and PGE2 augmented interleukin 1 beta (IL-1β)-mediated control of *M. tb* growth. Furthermore, patients with latent TB infection who successfully contained infection and did not develop active pulmonary TB appear to maintain balanced serum levels of PGE2 and LXA4 [30].

In addition to their cholesterol-lowering properties, statins also possess anti-inflammatory properties, which enhance their efficacy in the clinical management of atherosclerosis [31]. Statins can reduce IFN-γ-mediated MHC-II upregulation, and thus CD4 T cell activation – having clinical benefits in cardiac transplantation [32]. Statins may also downregulate TNF-α and IL-1β release via activation of the transcription factor Kruppel-like factor 2 (KLF2), as well as reduce IL-6, IL-8 and CCL5 secretion by myeloid cells and lymphocytes [31]. Prior use of statins has been shown to ameliorate inflammation-induced organ damage, thus reducing mortality among patients with sepsis and community-acquired pneumonia [33–36]. The therapeutic potential of statins has been evaluated in TB. Peripheral blood mononuclear cells derived from patients with familial hypercholesterolemia undergoing atorvastatin therapy were found to be more resistant to *M. tb* infection and replication [37]. It was established in a mouse model of TB that the cholesterol-lowering properties of atorvastatin potentiated this effect, where simvastatin treatment was concomitant with reduced pulmonary, hepatic and splenic *M. tb* burden and tissue pathology [37]. It was also shown that simvastatin potentiates autophagic cell death of *M. tb*-infected macrophages, thus having a dual approach in controlling TB disease. In a separate study, co-administration of simvastatin to an anti-TB chemotherapy regimen combining rifampicin, isoniazid and pyrazinamide led to improved bactericidal activity of the drugs against intracellular *M. tb* as well as in a mouse model of TB [38]. Clinically, patients with type 2 diabetes mellitus (T2DM) on statin therapy (as well as anti-diabetic therapy) seem to be at lower risk of developing active TB [39].

Metformin (MET) is an essential drug used in the treatment of T2DM, which acts by affecting the mitochondrial respiratory chain [40]. In a recent preclinical study, MET was evaluated as an adjunct therapeutic for drug-susceptible (DS)-TB and MDR-TB [41]. MET treatment induced superoxide anion production in macrophages infected with DS or MDR *M. tb* in an-adenosine monophosphate-activated protein kinase (AMPK)-dependent manner [41]. Furthermore, MET therapy potentiated the activity of the first-line anti-TB drugs isoniazid and ethionamide in a mouse model of TB, commensurate with improved anti-TB CD8 T cell responses, reduced concentrations of inflammatory cytokines and lung pathology [41]. A retrospective evaluation of patients with T2DM receiving MET therapy showed that they are less prone to develop cavitary pulmonary TB. In addition, T cell non-reactivity to ESAT-6 in a T-SPOT IFN-γ assay also suggested less incidence of latent TB infection among these individuals [41]. The ability of MET to promote maintenance of memory CD8 T cells via AMPK-driven fatty acid oxidation, as seen in mouse models of solid tumours [42, 43], may be an additional avenue for its potent immunomodulatory properties in TB.

Imatinib mesylate, a highly specific and successful drug that inhibits the Bcr-Abl tyrosine kinase in chronic myelogenous leukaemia has also been evaluated in TB [44]; treatment of *M. marinum*-infected mice with imatinib resulted in enhanced myelopoiesis in the bone marrow. Furthermore, an early influx of neutrophils into the lungs of infected mice helped reduce the bacterial burden. Imatinib in combination with rifampicin greatly reduced liver pathology in mice infected with *M. marinum*, while reducing lung bacterial load also in *M. tb*-infected animals [45].

The DNA alkylating agent cyclophosphamide (CP) is a potent anti-proliferative cancer drug and pre-conditioning agent metabolised by cytochrome P450 to produce reactive metabolites that crosslink with guanine residues [46, 47]. Cell types expressing low amounts of aldehyde dehydrogenase are most susceptible to CP treatment since its enzymatic activity neutralises drug-derived cytotoxicity [48]. Regulatory T cells (Tregs) express high cellular levels of ATP-binding cassette B1, which binds CP, thus making these cells especially vulnerable to the drug [49]. Conversely, aldehyde dehydrogenase overexpression can promote resistance to CP toxicity [50]. Prior CP administration to patients participating in a clinical trial of a renal cell cancer vaccine candidate (IMA901) led to Treg decline in peripheral blood of the participants, afforded better CD8 T cells priming and improved overall survival [51]. CP treatment may remove Treg cells as well as pathological lymphocytosis in patients with MDR-TB [52–54].

Histone deacetylase inhibitors (HDIs) comprise several clinically approved pharmacological agents which block the activity of the chromosome-modifying enzymes histone deacetylases to promote gene transcription [55]. Histone acetylation mediated by HDIs has been clinically beneficial in cancer therapy [56], vorinostat (suberoylanilide hydroxamic acid) was licensed in 2006 for the treatment of cutaneous T cell lymphoma [57], and valproic acid (VPA, currently in use for epilepsy and bipolar disorder) is in clinical trials for solid tumours [58], while many others are in late-stage clinical trials for a plethora of cancers [59]. VPA and vorinostat can induce apoptosis and autophagy in treated cells via oxidative burst and inhibition of the mammalian target of rapamycin (mTOR), respectively [60–62]. The effectiveness of HDIs in infectious disease has been evaluated against HIV infection and West Nile virus infection. VPA or vorinostat treatment of CD4 T cells from individuals latently infected with HIV-1 can reactivate viral progeny, making virus-infected cells highly susceptible to ART as well as CD8 T cell-mediated immune attack [63–66]. These observations have led to investigating the clinical efficacy of HDIs in HIV-infected individuals [67, 68]. In a mouse model of West Nile virus infection, co-treatment of vorinostat with an experimental antiviral drug candidate ameliorated brain inflammation and neuronal damage in addition to reduction in viral load [69]. Evaluation of phenylbutyrate (PBA), another HDI, against intracellular *M. tb* has been shown to potentiate pro-inflammatory signalling (upregulation of CASP1, IL12, and VDR genes, among others), antimicrobial peptide production, reduced *M. tb* survival, thus implying enhanced anti-TB T cell activity [70]. Clinical evaluation of PBA and vitamin D3 treatment in patients with pulmonary TB resulted in quicker sputum smear conversion and increased production of cathelicidin by peripheral blood monocyte-derived macrophages [71].

### Blockade of cytokine signalling

As previously mentioned, the uncontrolled release of pro-inflammatory cytokines, such as TNF-α, IL-6 and IFN-α/β, drives destructive immunopathology and severe lung disease in TB. The use of anti-TNF-α therapy (adalimumab) in a patient with severe pulmonary TB proved to be life-saving [72], and set the stage for anti-cytokine therapy in clinical management of TB. IL-6, a major player in exacerbating disease severity in patients with TB, is another potential candidate for clinical blockade [6, 73], further confirmed by reduction of IL-6 serum levels in TB patients post-treatment [74]. Preclinical evaluation of IL-6 receptor (IL-6R) blockade in a mouse model of TB improved specific anti-TB T cell responses in conjunction with less severe pathology and reduced *M. tb* burden in the lungs [75]. Monoclonal antibodies that inhibit the IL-6/IL-6R pathway are currently endorsed for treatment of immune-mediated diseases such as idiopathic juvenile arthritis and Castleman’s disease in humans (anti-IL-6, siltuximab; anti-IL-6R, tocilizumab) [76, 77], and are currently under investigation for cancer treatment [78]. Blockade of the IL-6/IL-6R pathway may, therefore, be considered for clinical management of severe pulmonary MDR-TB. Preclinical evaluation of bevacizumab (anti-vascular endothelial growth factor (VEGF) monoclonal antibody) in a rabbit model of TB showed that inhibition of VEGF-A was commensurate with modified angiogenesis in the granuloma, which improved penetration of a small molecule dye (representative of an anti-TB drug) as well as oxygenation [79]. Oehlers et al. [80] showed that VEGF promotes granuloma formation in *M. marinum*-infected zebrafish, while pharmacological inhibition of VEGF activity with pazopanib reduced neovascularisation as well as the mycobacterial burden in the animals. IL-4 is another cytokine associated with poor prognosis in clinical TB that quantitatively declines in response to anti-TB treatment [81–84]. A clinical trial investigating the adjunctive potential of IL-4 blockade (anti-human IL-4 monoclonal antibody, pascolizumab) in patients with DS pulmonary TB undergoing anti-TB treatment is currently underway (NCT01638520).

### Vitamin D3

Vitamin D3 (VD3) is important for host resistance to TB; pulmonary TB patients with VD3 deficiency are not able to mount adequate control of primary *M. tb* infection in the lung [85]. Further, VD3 has been implicated in immunological control of TB in humans involving orchestration of IFN-γ, IL-32 and IL-15 signalling [86, 87]. Recent evaluation of the therapeutic potential of VD3 in combination with PBA has shown improved anti-mycobacterial effects and faster conversion to AFB-negative sputum by the participating TB patients [71]. In vitro evaluation of *M. tb*-infected macrophages treated with VD3 resulted in upregulation of a collage of anti-inflammatory (IL-10, ARG1) and pro-inflammatory (IL1B, TNF) genes over a 72-hour exposure period [70] – this profile was relatively unchanged with PBA co-treatment. However, other clinical studies have not found a correlation between VD3 therapy and improved treatment outcome in TB patients [88, 89]. The therapeutic potential of VD3 in MDR-TB patients therefore warrants further clinical evaluation in larger cohorts of patients in endemic countries.

### Immune checkpoint inhibition

One of the most remarkable immunomodulatory therapies of recent times is currently licensed for melanoma treatment – antibody-based inhibition of the immune checkpoints programmed cell death 1 (PD-1) and cytotoxic T lymphocyte-associated antigen 4 (CTLA-4). This strategy activates and mobilises tumour-infiltrating T lymphocytes as well as those in peripheral blood of patients with metastatic melanoma [90]. Although immunologically purposed to regulate T cell responses for avoiding immune hyper reactivity, PD-1 and CTLA-4 expression on tumour-specific T cells abrogates anti-tumour effector functions and renders the T cells irresponsive [91]. Tregs have abundant expression of PD-1 on their surface; interaction of this molecule with its ligands (PD-L1/2) on target cells leads to expansion and dampening of antigen-specific effector T cell responses [92]. Importantly, a myriad of cytokines, including IFN-γ, TNF-α, type 1 interferons, GM-CSF, IL-2, IL-7 and IL-15, are actively involved in enhancing the expression of PD-1 on T cells and its ligands on antigen-presenting cells and tumour cells at sites of disease [93]. CTLA-4 binds to CD80 and/or CD86 to control the activation state of effector T cells in the aftermath of a successful immune response, and initiates tolerance by abrogating IL-2 production and downstream activation of signal transducer and activator of transcription 5 (STAT5) [94].

Results from clinical trials assessing monoclonal antibodies targeting PD-1 (pembrolizumab/nivolumab) and CTLA-4 (ipilimumab) in cancers other than melanoma, i.e., lung cancer and ovarian cancer, have been very promising [95, 96]. Anti-PD-1 therapy (nivolumab) has been fruitful also in treating patients with chronic hepatitis C virus infection, resulting in a dramatic reduction in viral load [97]. There are several ongoing clinical studies evaluating the efficacy of anti-PD-L1 therapy (avelumab, MEDI4736) in cancer (e.g., but not limited to, NCT02572843, NCT02584829, NCT02588131, NCT02088112). Several clinical studies have evaluated the role of the PD-1/PD-L1 pathway in the pathogenesis of TB. PD-L1, the main ligand for PD-1, is markedly expressed on human macrophages upon infection with virulent *M. tb* [98]. In vitro blockade of PD-L1 with monoclonal antibodies induced IFN-γ-triggered killing of the infected cells by autologous peripheral blood T cells from patients with pulmonary TB [98, 99]. Clinically, PD-1 expression on T cells declines over a course of standard anti-TB therapy – concordant with improved in vitro responsiveness of Th1 cells to antigenic stimulation [99, 100]. In addition, expression of PD-1 on the surface of neutrophils, Tregs and natural killer T cells indicates that destructive inflammation in TB patients may further impair the *M. tb*-specific immune response [53, 54, 101, 102]. Similar observations have been reported for CTLA-4 expression on Tregs and effector T cells isolated from peripheral blood of patients with active TB [53, 103, 104].

Lymphocyte-activation gene 3 (LAG3) is another immune checkpoint generally expressed by Tregs to modulate CD4+ T cell responses, and binds to MHC-II molecules with high affinity to induce T cell exhaustion several days after activation [105]. LAG3 was recently shown to be highly expressed in the lungs of macaques with active pulmonary TB and not in latent TB infection, suggesting a link between low LAG3 expression and successful containment of *M. tb* infection [106]. Combined blockade of PD-1 and LAG3 is able to rescue specific CD4+ and CD8+ T cell activity directed against *Plasmodium falciparum* in an experimental mouse model of malaria [95, 107]. Several clinical trials are currently registered for testing the efficacy of anti-LAG3 monoclonal antibodies (BMS-986016, GSK2831781, LAG525) in various cancers (NCT02061761, NCT01968109, NCT02195349, NCT02460224).

The fourth clinically relevant immune checkpoint molecule is T cell immunoglobulin and mucin domain 3 (TIM3). Immunologically, TIM3 binds to galectin 9 (Gal9) on the surface of antigen-presenting cells (as well as tumour cells) to induce apoptosis of activated T cells [108]. Inhibition of TIM3 (MBG453) in cancer therapy affords improvement of anti-tumour T cell responses, marked by IFN-γ secretion and CD8 T cell effector functions [108]. Pertinent to TB, in vitro TIM3 blockade in co-culture experiments with *M. tb*-infected macrophages from TB patients with or without HIV co-infection fostered bacterial killing and enhanced IL-1β secretion by infected cells as well as IFN-γ release by T cells [109, 110]. T cell-mediated reduction of bacterial burden in infected macrophages from HIV-TB patients was further improved in combination with anti-PD-1 blockade [110]. Along with PD-1 and CTLA-4, treatments targeting LAG3 and TIM3 may improve targeted immune responses in patients with MDR-TB for better clinical outcomes.

### Cellular therapy

In addition to chemical compounds, monoclonal antibodies and nutraceuticals, cell-based therapies have also been very promising in cancer and infectious disease [111, 112]. Adoptive transfer of tumour-infiltrating T lymphocytes has saved many lives in the last 20 years, with objective response rates reaching 56 % in patients with metastatic melanoma [113]. Transfer of donor-derived cytomegalovirus- and Epstein–Barr virus-specific CD8 T cells following stem cell transplantation is actively pursued in the clinic [111]. Pertaining to TB, re-infusion of bone marrow-derived mesenchymal stromal cells isolated from patients with MDR/extensively drug resistant-TB was shown to be safe [114], and a phase 2 trial is underway in Durban, South Africa, to evaluate their efficacy in improving treatment outcomes and effects on immune responses. Mesenchymal stromal cell treatment also re-programmed anti-TB T cell responses, with focus on specific *M. tb* antigens. Infusion of *M. tb*-specific T cells has yet to be clinically evaluated in TB patients, although in vitro evidence for feasibility, comprising subsets of CD8 T cells, NK T cells and TCR γδ T cells, has been established [115–119].

## Conclusion

The poor treatment outcomes of MDR-TB treatment using current WHO recommended TB drug treatment regimens now warrants newer approaches to therapy. The use of immunomodulatory agents as adjunct HDT for improving management outcomes warrants urgent evaluation in well designed, controlled randomized clinical trials. These studies will also provide opportunities to study the *M. tb*-specific immune responses in the lung and peripheral blood (B cells, T cells, macrophages, antibodies) as markers of inflammation, disease activity, cure or relapse. Well-designed, multicentre randomised clinical trials involving aptly defined patient cohorts are now required.
